# Outcomes of Perfused but Pulseless Versus Well-Perfused Pediatric Supracondylar Humerus Fractures Treated With Closed Reduction and Percutaneous Pinning

**DOI:** 10.7759/cureus.95736

**Published:** 2025-10-30

**Authors:** Syed Faisal Afaque, Nitin Jeenjwadia, Udit Agrawal, Vikas Verma

**Affiliations:** 1 Department of Paediatric Orthopaedics, King George's Medical University, Lucknow, IND

**Keywords:** closed reduction, pediatric elbow, percutaneous pinning, pink pulseless hand, supracondylar humerus fracture

## Abstract

Background: The management of pediatric supracondylar humerus fractures (SHF) with a pink pulseless hand (PPH) remains controversial. Traditional teaching advocated urgent vascular exploration, but many centres now favour closed reduction and percutaneous pinning (CRPP), followed by observation if the hand stays warm and well-perfused.

Objective: This study aimed to compare clinical and radiological outcomes of displaced pediatric SHF in two groups: those presenting with a PPH versus those with a well-perfused hand and palpable pulse. All patients were managed with CRPP.

Methods: We retrospectively reviewed cases at King George’s Medical University, a tertiary pediatric orthopaedic centre in India, from January 2018 to December 2023. Children ≤12 years old with closed or Gustilo-Anderson type I open displaced SHF (Gartland type III or IV) treated by CRPP were included. Patients with neurologic deficits, those requiring open reduction, or those undergoing vascular exploration were excluded from the study. Group I (n = 18) presented with a PPH (absent radial pulse, but a pink, warm hand with brisk capillary refill and normal oxygen saturation); brachial artery continuity was confirmed by duplex ultrasound. Group II (n = 24) presented with a well-perfused hand with a palpable radial pulse. All patients underwent CRPP under fluoroscopic guidance. Two lateral K-wires (with an additional wire if needed for stability) were used, and an above-elbow cast was applied in 50-60° flexion. Vascular status was reassessed intra- and postoperatively. Follow-up visits at three, six, and 12 weeks included clinical exams and radiographs. Outcomes evaluated were Flynn’s criteria, Baumann’s angle, maintenance of reduction, elbow range of motion (ROM), carrying angle, time to union, and final vascular status.

Results: A total of 42 children (mean age 7.5 years, 62% boys) were analysed. The two groups were similar in age, sex, affected side (60% left), and fracture patterns (mostly extension-type with posterolateral displacement). The mechanism of injury differed: Group I experienced more falls during play, whereas Group II experienced more falls from heights (p = 0.05). According to Flynn’s criteria, excellent or good functional outcomes were achieved in 94-96% of cases in both groups (77.8% excellent in Group I vs. 79.2% in Group II, plus 16.7% good in each; p = 0.97). Postoperative Baumann’s angles were comparable; the mean change in angle from immediately post-op to final follow-up was 1.89° in Group I and 2.25° in Group II (p = 0.72). The final carrying angle was slightly smaller in Group I on the right side (10.3° vs. 11.79° in Group II, p = 0.03), but this ~1.5° difference was not clinically significant. Elbow ROM recovered similarly in both groups: by 12 weeks, all patients attained ≥120° of flexion (p = 0.96). By the final follow-up (nine months), every child had a palpable radial pulse and normal perfusion; none reported cold intolerance.

Conclusions: CRPP yields excellent and comparable functional outcomes in pediatric SHF, regardless of whether the radial pulse is initially present, as long as the hand is well perfused. Routine emergent brachial artery exploration is not indicated for most PPHs after fracture reduction, provided that perfusion remains intact and vigilant monitoring is in place. These findings support a conservative observational approach to the PPH in SHF, reserving open vascular surgery for cases that develop signs of ischemia.

## Introduction

Supracondylar humerus fractures (SHFs) are among the most common traumatic elbow fractures in children, accounting for up to 60% of pediatric elbow fractures [1]. The peak incidence is between five and seven years of age, and the non-dominant limb is affected in the majority of cases [1]. Most SHFs result from a fall on an outstretched hand and are extension-type injuries (posterior displacement) in approximately 97% of cases [1]. Gartland’s classification is used to describe these fractures on coronal plane radiographs [2]. Type I fractures are nondisplaced or minimally displaced (intact anterior humeral line), type II are angulated with cortical contact, and type III are completely displaced [3]. An additional type IV (multidirectional instability) was later defined by Leitch et al. [4]. Gartland type I and II fractures can often be treated conservatively with casting, whereas type III and IV injuries typically require surgical fixation; a recent comparative study found that outcomes are equivalent when treatment is appropriate to the fracture’s severity [5,6].

Besides the bony injury, SHFs can be associated with neurovascular complications. Neuropraxia of the median, anterior interosseous, ulnar, or radial nerves may occur and usually recover spontaneously. Vascular compromise is a serious concern, often indicated by an absent pulse with signs of ischemia (pale colour, cool skin, delayed capillary refill) or, in some cases, a well-perfused pulseless hand [7,8]. A “pink pulseless hand” (PPH) refers to an extremity that lacks a palpable radial pulse but remains warm with normal capillary refill and a viable appearance [7]. This presentation raises a management dilemma: urgent vascular exploration versus careful observation.

For displaced (Gartland types II, III, and IV) supracondylar fractures, the standard treatment is prompt closed reduction and percutaneous pinning (CRPP) under anaesthesia [9]. In a pale, pulseless hand (cool, poorly perfused), there is consensus that emergent open reduction of the fracture and brachial artery exploration are required [9]. However, in cases of a PPH, management is controversial. Some clinicians still advocate immediate exploration to avoid an occult brachial artery injury and potential ischemia [10,11]. Increasing evidence, however, suggests that if the hand remains well perfused after fracture reduction, vigilant observation may be a safe approach, as many will spontaneously regain a pulse without surgical vascular intervention [12]. Recent reports have documented favourable outcomes in perfused pulseless cases managed with CRPP and monitoring, with low rates of late ischemic complications [13]. To date, few studies have directly compared outcomes of PPHs to those of “pulsed” hands in the context of pediatric SHF. The purpose of this study was to evaluate whether the absence of an initial pulse (with adequate perfusion) affected the results of CRPP treatment. We hypothesised that functional and radiological outcomes would be equivalent between children with a well-perfused pulseless hand and those with a normal pulse, provided perfusion was maintained.

## Materials and methods

Study design

We conducted a retrospective cohort study at the Department of Pediatric Orthopaedics, King George’s Medical University (KGMU), a high-volume pediatric orthopaedic centre in North India, after obtaining Institutional Review Board approval (IEC/907/Ethics/2024). The study period spanned from January 2018 to December 2023. Clinical and radiographic data were retrieved from hospital records and radiology archives.

Participants

Inclusion criteria were children aged ≤12 years with a displaced SHF (Gartland type III or IV) that was closed or a Gustilo-Anderson grade I open fracture. All included fractures were treated with closed reduction and percutaneous pinning (CRPP). Two patient groups were defined based on vascular status at presentation:

Group I (PPH) included children presenting with a PPH, i.e., absent palpable radial pulse, but hand warm with brisk capillary refill (<3 seconds) and oxygen saturation (by pulse oximeter) equal to the contralateral hand. In these patients, continuity of the brachial artery was confirmed on colour Doppler duplex ultrasound.

Group II (pulsed hand) included children presenting with a well-perfused hand and a palpable radial pulse.

Exclusion criteria included any patient with signs of an ischemic (“white”) hand requiring immediate open exploration, fractures that underwent primary open reduction or any vascular repair procedure, and cases with pre-operative neurological deficits. Patients lost to follow-up before 3 months or with incomplete records were also excluded.

Treatment protocol

All patients were promptly taken for CRPP under general anaesthesia after initial assessment and stabilisation. The injured elbow was gently reduced by traction, correction of any medial/lateral translation and hyperextension, and then flexion, with reduction confirmed under fluoroscopy. Stabilisation was achieved with two smooth Kirschner wires (K-wires) inserted percutaneously from the lateral condyle across the fracture. The pins were placed based on the fracture pattern and surgeon preference. If the fracture remained unstable on fluoroscopic stress testing [13], or in certain Gartland type IV patterns, a third K-wire was added (either an additional lateral wire or a medial wire, as needed, while taking care to protect the ulnar nerve). All pins were cut and bent outside the skin. After pinning, the extremity was immobilised in an above-elbow splint with the elbow flexed about 50-60°.

Immediately after reduction and pinning, we checked for the return of the radial pulse. If the pulse was still absent, we confirmed that the hand remained warm with normal capillary refill and used a Doppler device to detect radial and ulnar arterial signals. In no case in this series was an immediate open exploration performed, since all pulseless hands were well perfused (by our Group I definition). After the slab application, the compartment status was assessed clinically, and the child’s hand was observed for adequate perfusion.

Postoperative care

The children were admitted for at least 48 hours of observation with serial neurovascular checks. The operated limb was kept elevated, and finger ROM exercises were encouraged immediately. Pulses (by palpation or Doppler) and perfusion (colour, temperature, capillary refill) were documented hourly for the first 24 hours. Parents were educated on signs of vascular compromise (excessive pain, pallor, coolness, swelling) prior to discharge. Patients were typically discharged on postoperative day 3 after the first dressing change, provided the hand remained warm and the child was otherwise stable.

Outpatient follow-up visits were scheduled at three weeks (for X-ray and pin removal), six weeks, and 12 weeks. All follow-ups were conducted by the senior surgeon using a standardised protocol. At three weeks, once early healing was evident on radiograph, the K-wires were removed in the clinic, and the child began gentle active elbow mobilisation. Clinical exams at follow-up assessed carrying angle, elbow ROM (using a goniometer), and any neurologic deficits. Radiographic outcomes were measured on anteroposterior (Baumann’s angle) and lateral (anterior humeral line) elbow X-rays.

Outcome measures

Primary Functional Outcome

The primary functional outcome was graded by Flynn’s criteria at final follow-up (12 weeks) [10]. This system grades results as excellent, good, fair, or poor based on the loss of carrying angle and loss of elbow motion [10]. “Excellent” corresponds to ≤5° loss of carrying angle and motion, “good” for ≤10°, “fair” for 11-15°, and “poor” for >15° loss [10].

Secondary Outcomes

Carrying angle: It is measured clinically (with the elbow extended) in both arms at the final follow-up to detect any change (i.e., cubitus varus/valgus deformity compared to the uninjured side).

Baumann’s angle: It is measured on the AP radiograph immediately after surgery and at 12 weeks. The change in Baumann’s angle between these time points was used to evaluate the maintenance of reduction. We classified loss of reduction according to the criteria of Skaggs et al. [14]: <6° change (none), 6-12° (moderate), and>12° (significant loss).

Elbow ROM: Specifically, the flexion arc at 12 weeks was recorded. We categorised the final ROM for comparison as follows: 0-125°, 125-130°, 130-135°, and > 135°.

Time to union: It is defined as radiographic healing (bridging callus and indistinct fracture line) accompanied by the restoration of cortical continuity.

Vascular status at the final follow-up: It notes whether the radial pulse was palpable and any reports of cold intolerance or hand claudication during activity.

Statistical analysis

Data was compiled in Microsoft Excel (Microsoft Corp., USA) and analysed using IBM SPSS Statistics for Windows, Version 25.0 (released 2017, IBM Corp., Armonk, NY). Continuous variables were described as mean ± standard deviation and compared between groups using the independent samples t-test. Categorical variables were compared using Chi-square or Fisher’s exact test as appropriate. A p-value < 0.05 was considered statistically significant.

## Results

Patient cohort

During the six-year study period, 937 pediatric elbow injuries were treated at our centre, of which 106 were supracondylar humerus fractures. Applying the inclusion/exclusion criteria, 42 cases were eligible for analysis. Group I (pink pulseless hand) consisted of 18 children, and Group II (well-perfused hand with pulse) consisted of 24 children.

Demographics

The groups were comparable in baseline characteristics (see Table 1). The mean age was 7.5 years (6.8 in Group I vs 7.9 in Group II). Boys comprised about two-thirds of each group. The right side was dominant in most children (~80%), but the left arm was fractured more often in both groups (~60% of cases). The injury was extension-type in all patients; 79-83% of fractures were displaced posterolaterally, while the rest were displaced posteromedially. The mechanism of injury showed a notable difference: Group I injuries most commonly occurred from a fall while playing on the ground (~44% of cases), whereas Group II injuries more often resulted from a fall from a height (~71% of cases). This discrepancy in mechanism approached statistical significance (p = 0.05). Four injuries in Group I and two in Group II were due to road traffic accidents.

**Table 1:** Demographic and injury characteristics of Groups I and II (perfused pulseless vs. pulsed hand)

	Group I (N = 18)	Group II (N = 24)	P-value
Mean age (years)	6.83 ± 2.33	7.92 ± 2.39	0.14
Sex (M: F)	12 (66.7%): 6 (33.3%)	15 (62.5%): 9 (37.5%)	1.0
Dominant hand (R: L)	15 (83.3%): 3 (16.7%)	18 (75.0%): 6 (25.0%)	0.78
Fracture side (R: L)	7 (38.9%): 11 (61.1%)	9 (37.5%): 15 (62.5%)	1.0
Mechanism of injury			
Fall from height	6 (33.3%)	17 (70.8)	0.05
Fall while playing	8 (44.4%)	5 (20.8%)	
Road traffic accident	4 (22.2%)	2 (8.3%)	
Fracture displacement			
Posteromedial	3 (16.7%)	5 (20.8%)	1.0
Posterolateral	15 (83.3)	19 (79.2%)	

Operative findings

Closed reduction under fluoroscopy was successful in all 42 cases; none required open reduction. Intraoperatively, all fractures were stabilised with lateral-entry pinning (two wires in 36 cases, three wires in six cases due to either instability or a type IV pattern). After reduction and casting, seven of the 18 PPH cases regained a palpable radial pulse in the operating room. The remaining 11 PPH cases were confirmed by Doppler to have flow signals (although no palpable pulse) and continued to have a warm, pink hand. All 24 children in Group II had a palpable pulse after reduction. There were no instances of compartment syndrome.

Follow-up outcomes

The key postoperative outcomes are summarised in Table 2. All fractures achieved solid union by 12.6 weeks in Group I and by 12.9 weeks in Group II, on average, with no significant difference noted between the two groups (p = 0.85). Alignment was maintained in both groups: according to the criteria of Skaggs et al., 89% of Group I and 92% of Group II had no significant change (<6° shift) in Baumann’s angle from immediate post-op to final follow-up, and only one patient in each group had a moderate change (~7°) without clinical consequence. No case in either group had a loss of alignment greater than 12 ° or required re-manipulation.

**Table 2:** Comparative clinical and radiological outcomes of Groups I and II (perfused pulseless vs. pulsed hand)

	Group I (N = 18)	Group II (N = 24)	P-value
Carrying angle at 12 weeks			
Right arm	10.33° ± 2.95°	11.79° ± 1.17°	0.03
Left arm	11.56° ± 1.38°	10.96° ± 1.78°	0.24
Difference (injured vs. normal)	1.78° ± 2.66°	1.08° ± 1.53°	0.65
Baumann’s angle (post-op)			
Right side	76.22° ± 2.75°	77.00° ± 2.85°	0.38
Left side	75.25° ± 3.67°	76.22° ± 3.35°	0.90
Change (post-op to 12 weeks)	1.89° ± 2.32°	2.25° ± 2.72°	0.72
Elbow flexion at 12 weeks			
<125°	4 (22.2%)	4 (16.7%)	0.96
125–129°	3 (16.7%)	5 (20.8%)	
130–134°	7 (38.9%)	9 (37.5%)	
≥135°	4 (22.2%)	6 (25.0%)	
Flynn grade (12 weeks)			
Excellent	14 (77.8%)	19 (79.2%)	0.97
Good	3 (16.7%)	4 (16.7%)	
Fair	1 (5.6%)	1 (4.2%)	
Poor	0	0	
Follow-up duration (months)	9.39 ± 1.68	8.71 ± 1.36	0.18
Time to union (weeks)	12.6 ± 1.1	12.9 ± 1.3	0.43
Loss of reduction (in degree)	3.33° ± 4.85°	3.13° ± 4.61°	0.89
Complications (pin site infection)	1 (5.55%)	1 (4.16%)	1.0

Loss of carrying angle (cosmetic outcome) and final radiographic alignment were also similar between groups. The final carrying angle at three months averaged ~11° in both groups (measured on the uninjured side vs the injured side), with no statistical difference except for a slight between-group difference on the right side (mean 10.3° in Group I vs. 11.8° in Group II, p = 0.03). This ~1.5° difference in carrying angle is not clinically meaningful.

Functional results by Flynn’s grading were essentially equivalent. In Group I, 14 patients (77.8%) had an “excellent” result and three (16.7%) “good,” with 1 “fair” (5.6%, who lacked ~20° of flexion). In Group II, 19 patients (79.2%) were excellent, four (16.7%) were good, and one (4.2%) was fair. There were no “poor” outcomes in either group. The distribution of Flynn grades showed no statistical difference (p = 0.97). The two fair outcomes (one in each group) corresponded to the two cases with mild persistent flexion loss at the elbow; these patients had 10-20° less flexion than the uninjured side at three months but improved to full range by six months.

Elbow ROM recovery was alike in both cohorts. By 12 weeks post-op, the vast majority of children in each group had regained nearly full flexion. Specifically, about 61% of Group I and 62.5% of Group II could flex the elbow ≥130° (with full extension), and only 22% of Group I and 16.7% of Group II had final flexion <125°. There was no significant difference in the overall ROM category distribution (p = 0.96). Nearly all patients in both groups achieved a functional arc of motion (within 15° of full extension to at least 130 ° of flexion) by six months.

**Figure 1:**
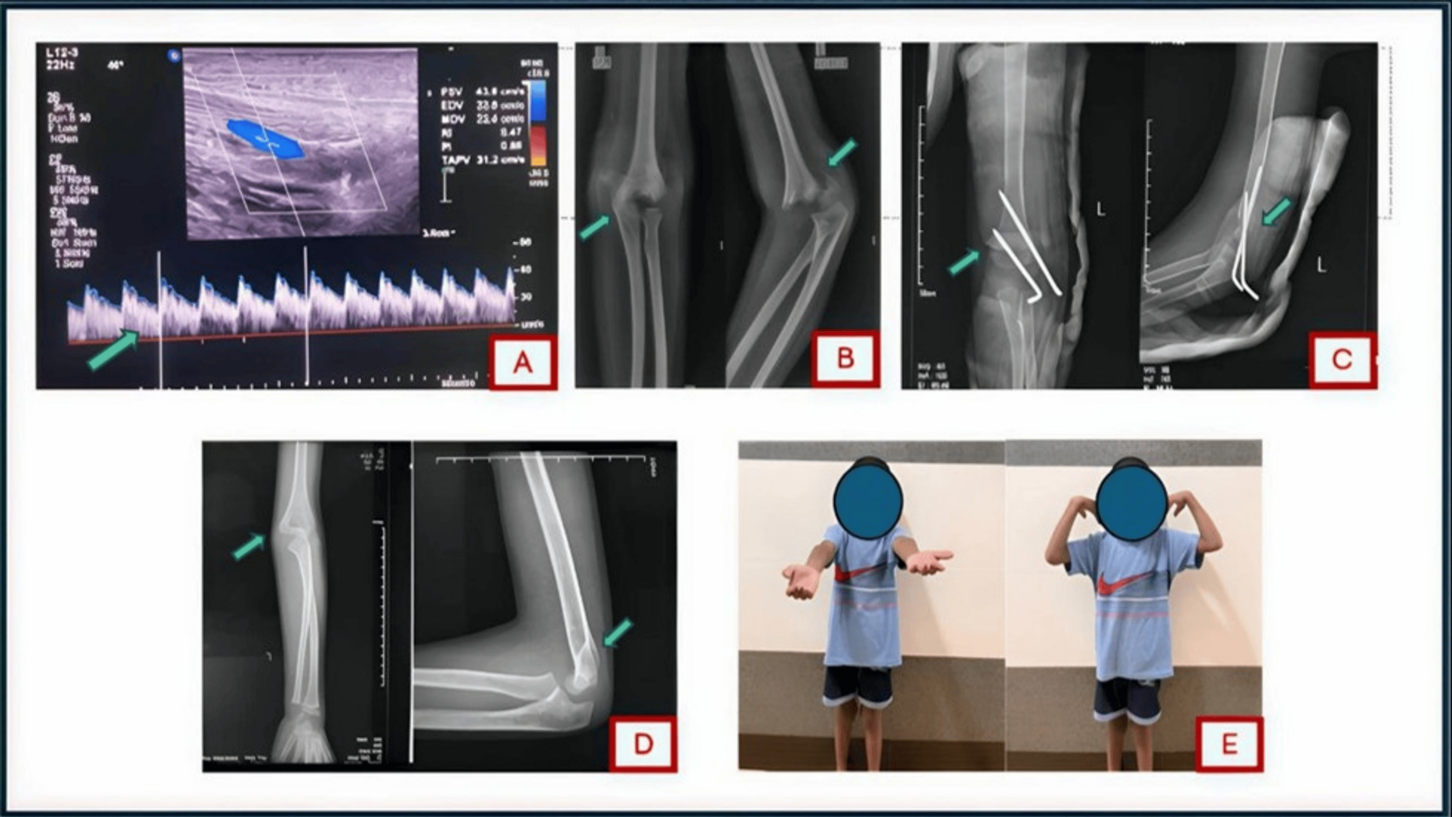
A nine-year-old boy with a Gartland type III displaced supracondylar humerus fracture presenting with a pink pulseless hand. (A) Color flow duplex ultrasound of the brachial artery shows (arrow) loss of the normal triphasic waveform (converted to a monophasic flow), indicating a brachial artery injury with maintained collateral perfusion. (B) Pre-reduction anteroposterior (AP) and lateral radiographs of the elbow demonstrating the completely displaced fracture (arrows). (C) Immediate post-reduction radiographs after closed reduction and percutaneous pinning, showing restoration of alignment (arrows). (D) Radiographs at 12 weeks post-operatively confirming successful fracture union (arrows) in good alignment. (E) Clinical photograph at 12 weeks demonstrating the patient’s regained full elbow range of motion.

**Figure 2:**
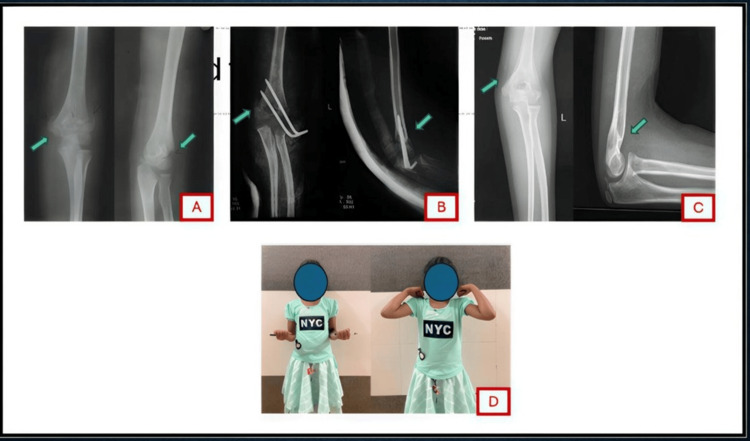
A seven-year-old girl with a Gartland type III displaced supracondylar humerus fracture and a well-perfused hand with palpable pulse at presentation. (A) Pre-reduction AP and lateral radiographs showing the displaced supracondylar fracture (arrows). (B) Post-reduction radiographs after CRPP, showing anatomic realignment of the fracture (arrows). (C) Radiographs at 12 weeks post-op demonstrating solid union of the fracture (arrows) with maintained alignment and carrying angle. (D) Clinical image at 12-week follow-up illustrating full functional recovery of elbow motion.

Importantly, vascular outcomes were uniformly positive. By the six-week visit, all 18 patients with PPH had regained a palpable radial pulse. In seven of those, the pulse returned within 24 hours postoperatively; in another six, it returned by three weeks; and in the remaining five, it returned by six to 12 weeks. At final follow-up (~9 months), every child in both groups had a normal palpable pulse and a well-perfused hand. We observed no cases of claudication, cold intolerance, or limb length discrepancy. Two children (one in each group) developed a superficial pin tract infection during casting; these resolved with local dressings and oral antibiotics. No other complications were noted. Case examples of Group I and Group II are shown in Figure 1 and Figure 2, respectively.

## Discussion

This study demonstrates that a perfused but pulseless hand in pediatric supracondylar fractures can be managed successfully with closed reduction and pin fixation, yielding outcomes comparable to those with an intact pulse. All 18 patients with an initially pulseless hand eventually recovered a palpable pulse, and their functional results were equally as good as those of children who had palpable pulses from the outset. These findings support the growing consensus that routine vascular exploration is not necessary in a PPH after fracture reduction, as long as the hand remains well perfused [11,12].

The ideal management of the PPH has been debated. On one side, some clinicians argue for immediate surgical exploration of the brachial artery in all pulseless cases, citing the risk of a significant arterial injury or thrombosis that could lead to ischemia [5,8]. Studies have shown that in perfused yet pulseless limbs, a majority (approximately 70-80%) have a brachial artery lesion (entrapment, intimal tear, or occlusion) [5,6]. Proponents of early exploration note that repairing such an injury can restore flow and potentially prevent long-term complications, and they report high arterial patency rates after timely repair [8]. Noaman et al. reported successful microsurgical reconstruction of brachial artery injuries in children with SFH and absent pulses [8]. Likewise, White et al. performed a systematic review and found that the absence of a pulse usually signifies an arterial injury even if the hand is pink; they suggested more aggressive evaluation and intervention in selected cases [5]. Mangat et al. analysed children with a “PPH” and found that those with concomitant median nerve palsy had a high likelihood of brachial artery entrapment; they recommended early exploration if a nerve deficit is present alongside pulselessness [6]. Our protocol differed in that we did not routinely explore any PPH case immediately. Notably, none of our PPH patients had an early neurologic deficit suggestive of vessel entrapment, which may partly explain why observation was successful in all cases.

On the other side of the debate, many authors, including the present study, support a more conservative approach to managing a well-perfused pulseless hand after CRPP [11,12]. The rationale is that perfusion (warmth and capillary refill) is the critical indicator of limb viability; if it remains normal, the collateral circulation around the elbow is maintaining hand perfusion despite the brachial artery injury or spasm [11,13]. Several studies have documented that a substantial proportion of perfused pulseless cases will spontaneously regain their pulse over time, and even those who do not will often develop sufficient collateral circulation without functional deficit [11]. In our series, 100% of PPH patients recovered a pulse within weeks, and none showed exercise intolerance or growth issues, echoing those reports. Pirone et al. similarly noted that vascular compromise in pediatric SHF can often be managed by fracture reduction alone, without the need for vascular surgery [13].

Multiple recent studies of perfused, pulseless SHF have shown excellent outcomes with CRPP and observation. For instance, Muslu et al. reported 92% excellent results (as per Flynn’s criteria) in a cohort of 100 completely displaced SHF treated with CRPP; notably, they excluded cases with vascular injury [7]. Rengarajan et al. also found predominantly excellent functional outcomes in Gartland type III fractures managed with CRPP, without routine vascular explorations [15]. Furthermore, Afaque et al. [16] and Kocher et al. [17] both found that different pinning techniques (crossed pins vs. lateral-only pins) did not significantly affect Baumann’s angle or functional outcomes, reinforcing that achieving a stable closed reduction is the key factor. These studies underscore that CRPP is an effective treatment for these injuries.

Notably, Shah et al. performed a focused literature review on the PPH and concluded that vascular exploration may be unnecessary if the hand remains well perfused after reduction [9]. Our real-world data strongly supports that conclusion. All our PPH patients did well with watchful waiting; none developed ischemia. It appears that in many cases of PPH, the brachial artery, while narrowed or kinked, continues to supply sufficient blood via collaterals until it recanalises or until flow improves as swelling subsides. Consistent with these findings, Badkoobehi et al. also recommended close observation (with the fracture reduced) for a PPH, reserving arterial exploration for signs of threatened perfusion [18].

It is important to emphasise patient selection and vigilant monitoring. The conservative approach for PPH is only safe if strict vigilance is maintained. We admit PPH patients for at least 48 hours of hospital observation with serial exams. If, at any point, the hand’s perfusion were to deteriorate (becoming cool, capillary refill time>2-3 seconds, progressive pain/swelling, or new neurologic signs), we would promptly proceed to exploration. In this series, no such deterioration occurred once the fracture was stabilised-a finding echoed by Ramesh et al. [11], who observed seven children with PPHs and reported spontaneous pulse return in all without the need for surgery. They recommended a “watchful expectancy” for a well-perfused pulseless hand, reserving surgical exploration only for cases of worsening pain (e.g., increasing forearm pain beyond 12 hours) or neurologic deterioration. Our experience aligns with that recommendation.

Some have raised concerns about the long-term vascular integrity in these cases, for example, whether an intimal arterial injury could predispose to late thrombosis or insufficient collateral circulation. In intermediate-term follow-ups (e.g., 9-12 months, as in our study), such problems appear to be rare. Scannell et al. [19] provided an intermediate-term follow-up of children with perfused, pulseless SHF and found that after an average of ~20 months, all had a palpable distal pulse, normal arm growth, and good/excellent function, even though 25% of those patients had a persistent brachial artery occlusion on imaging. In our study, we did not perform routine late imaging of the brachial artery; however, clinical exams were sufficient to ensure that there was no ischemia. None of our patients had exercise-induced symptoms or temperature intolerance, which implies adequate circulation (whether through a recanalised artery or robust collaterals).

Strengths and limitations

The strengths of this study include a homogeneous cohort from a single institution with a uniform treatment protocol and follow-up regimen. By directly comparing PPH and pulsed-hand groups with the same management, we controlled for treatment variability. We also used objective criteria (Flynn’s grades, Baumann’s angle change) to assess outcomes. The main limitation is the relatively modest sample size, especially for the PPH group (n = 18). However, PPH was an infrequent presentation (~17% of displaced SFH in our series), and our cohort size is comparable to that of other reports on this topic. The retrospective design is another limitation; although data were recorded prospectively in clinical notes, the study was not randomised. There is also an inherent selection bias in that only perfused pulseless cases were observed - a child with any signs of ischemia would have been explored and thus excluded. Finally, long-term vascular status beyond one year was not assessed (e.g. by angiography); nevertheless, all patients were asymptomatic at the latest follow-up.

## Conclusions

When treating a child with a displaced supracondylar humerus fracture, the absence of a radial pulse does not necessarily preclude an excellent outcome, provided that the hand remains well perfused. In our experience, CRPP, with careful observation, was successful in all cases of pink pulseless patients. As long as the hand is warm and has brisk capillary refill, immediate vascular exploration may not be necessary. Instead, one can proceed with fracture reduction and fixation and closely monitor perfusion. A prompt pulse return is common within hours to weeks. If perfusion deteriorates at any point, vascular surgery can be performed at that time. This selective approach can avoid many unnecessary surgical explorations while still ensuring limb safety. Our findings contribute to the growing evidence that a well-perfused pulseless hand can be managed conservatively with vigilant monitoring, reserving arterial repair for rare cases that demonstrate threatened circulation.
